# Supplementary material for: Multi-Strain Probiotic Supplementation with a Product Containing Human-Native S. salivarius K12 in Healthy Adults Increases Oral S. salivarius
Source: Nutrients. 2021 Dec 8;13(12):4392. doi: 10.3390/nu13124392 (PMC8707698; doi:10.3390/nu13124392)
Supplement: Supplementary file 1 [file nutrients-13-04392-s001.zip › nutrients-1465373-SM.pdf]

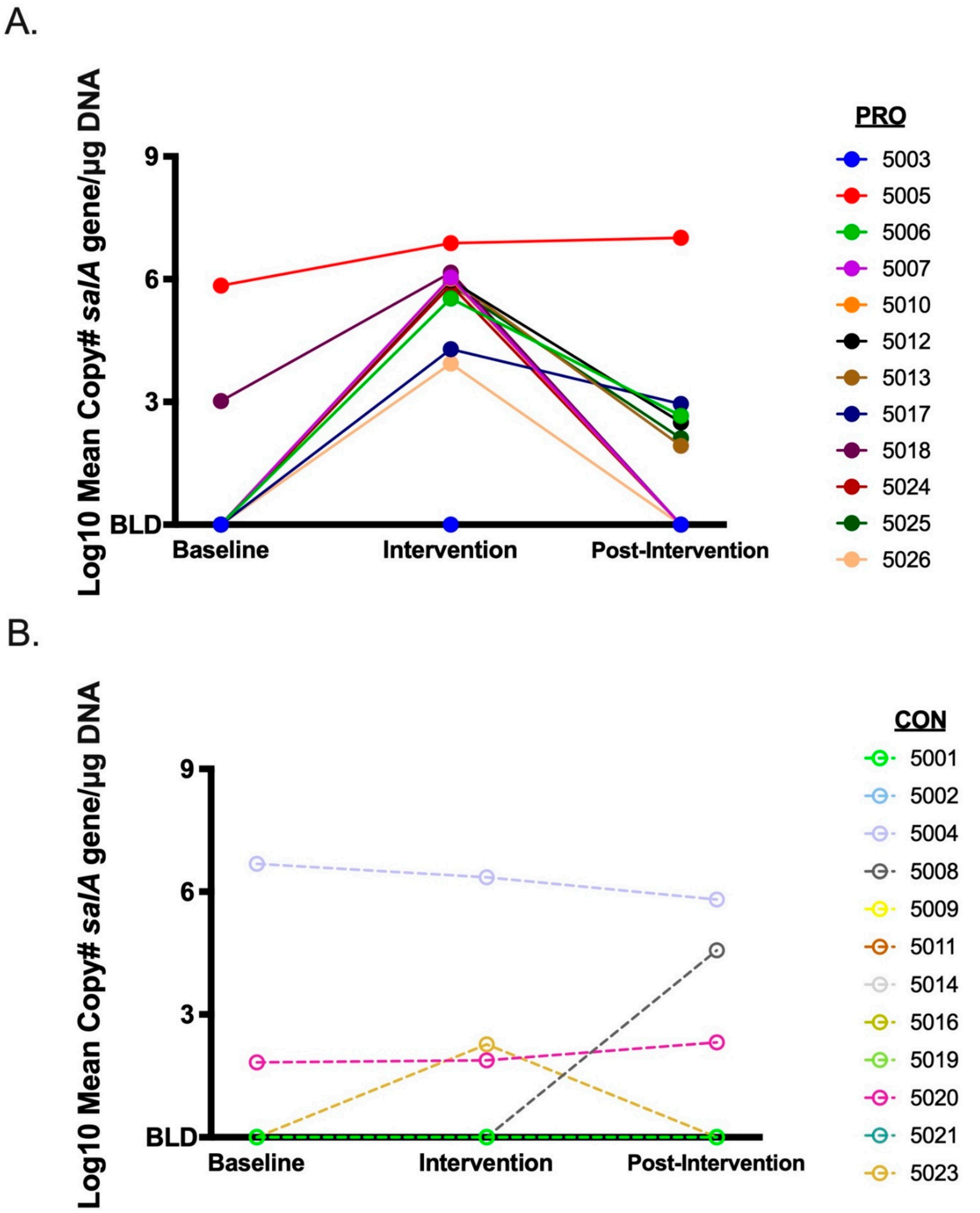

Figure S1. Salivary *S. salivarius* over the study period for each participant
A. Probiotic (PRO) intervention. B. Control (CON) intervention. Below detection limit, BLD.

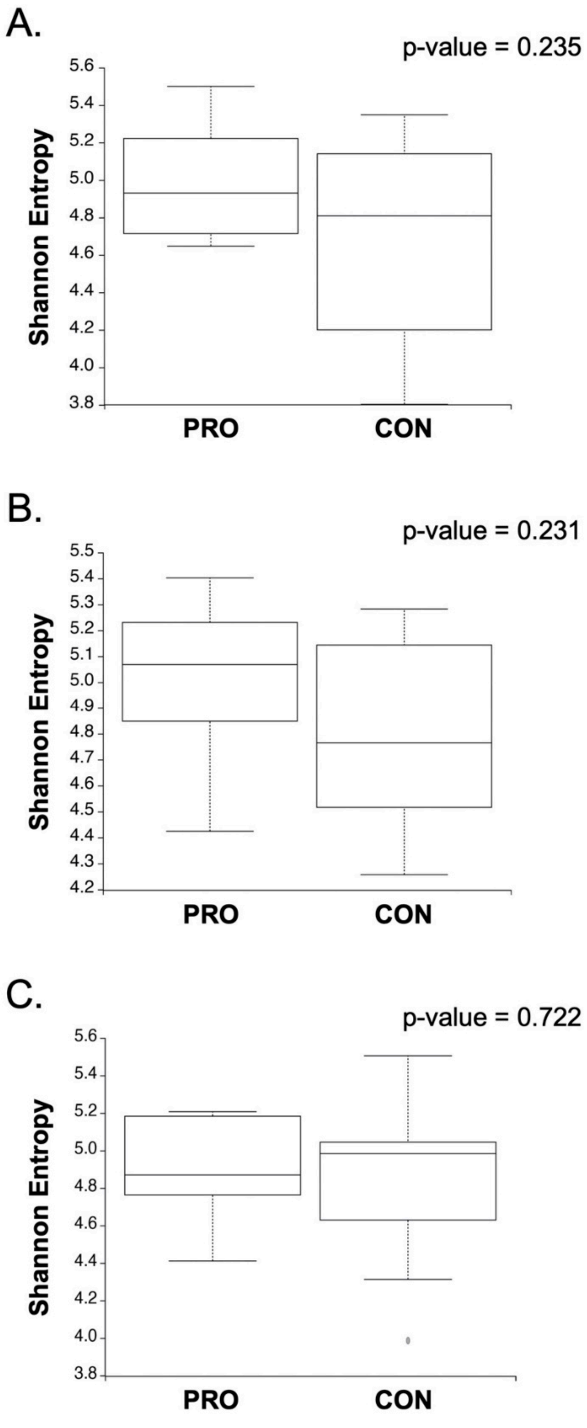

Figure S2. Alpha diversity between groups over the study period
Alpha diversity boxplots showing Shannon richness of the microbial communities separated by treatment PRO and CON. Differences between alpha diversity of the different treatment groups was calculated using Kruskal-Wallis pairwise testing between groups. A. Baseline. B. Intervention. C. Post-Intervention

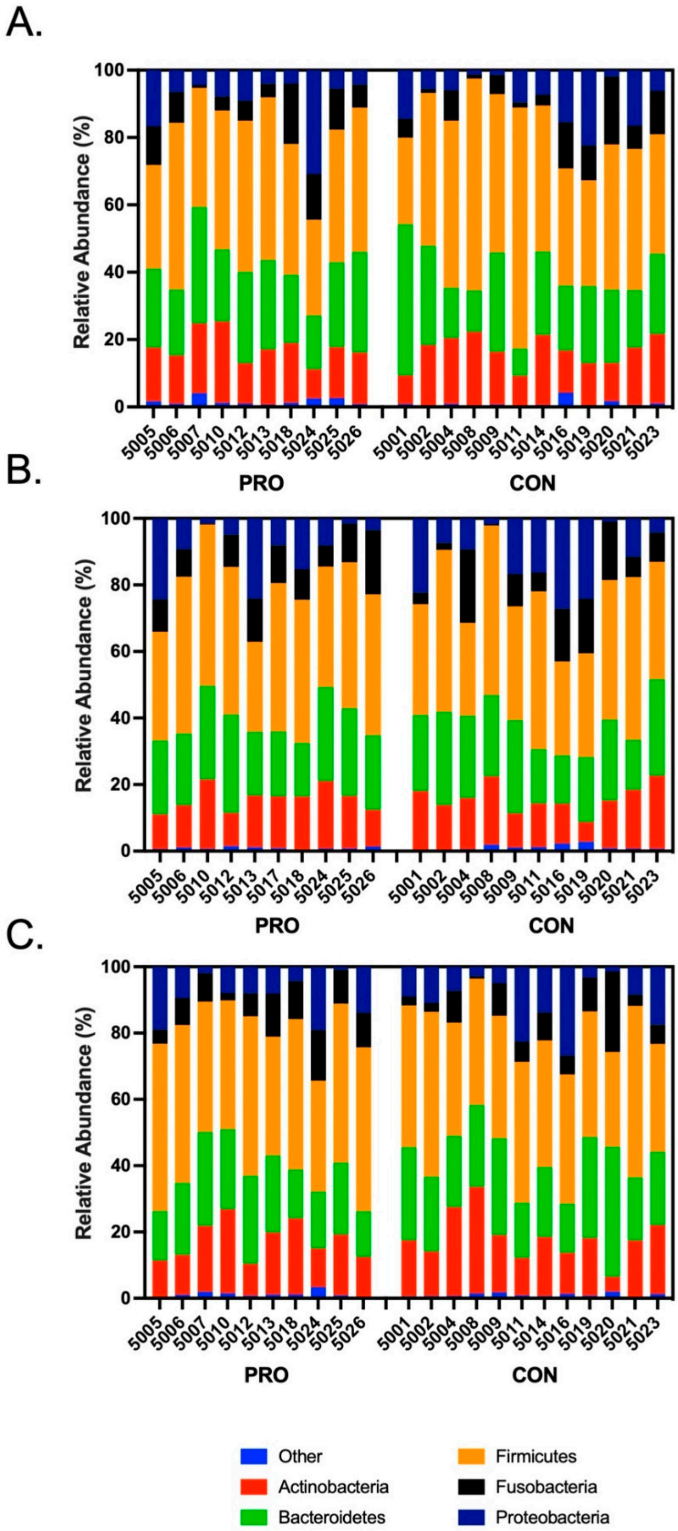

Figure S3. Salivary microbiome relative abundance on a phyla level
Thirteen phyla were detected in all samples across all time points. The bar graphs

represent the 5 phyla present at 10% relative abundance or greater; all phlya less than 10% are represented as “other” A) Baseline; B) Intervention; C) Post-intervention. Data are per-protocol with participant 5003 removed from the analysis. Additionally, data were not available for one other participant in the PRO group.

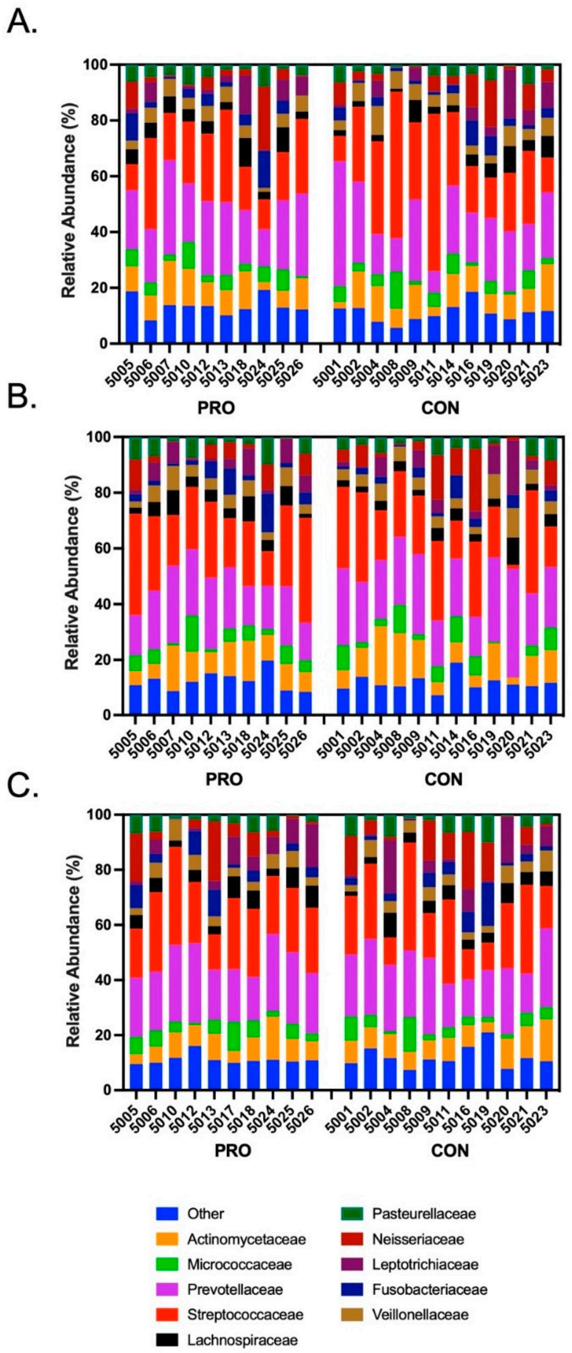

Figure S4. Salivary microbiome relative abundance on a family level
Ninety-three families were detected in all samples across all time points. The bar graphs represent families present at greater than 10% relative abundance. Families present at

less than 10% relative abundance are represented as “other. A) Baseline; B) Intervention; C) Post-intervention. Data are per-protocol with participant 5003 removed from the analysis. Data are not available for one additional participant in the PRO group and one participant in the CON group due to low amplicon sequence variants counts.

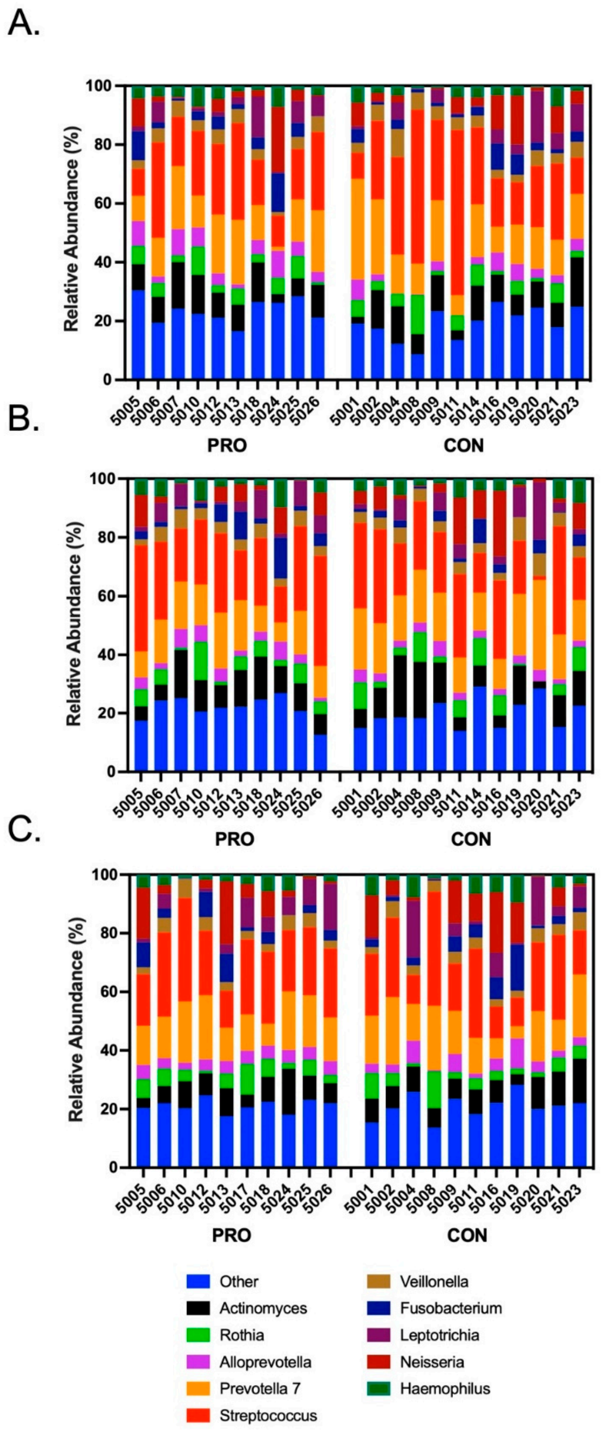

Figure S5. Salivary microbiome relative abundance on a genus level
Two hundred fifty-three genera were detected in all samples across all time points. The bar graphs represent all genera detected, but the ten genera with relative abundance greater than 10% in any of the samples are represented in the key; all other genera are represented as “other”. A) Baseline; B) Intervention; C) Post-intervention. Data are per-protocol with participant 5003 removed from the analysis. Data are not available for one participant in the PRO group and one participant in the CON group due to low amplicon sequence variants counts.

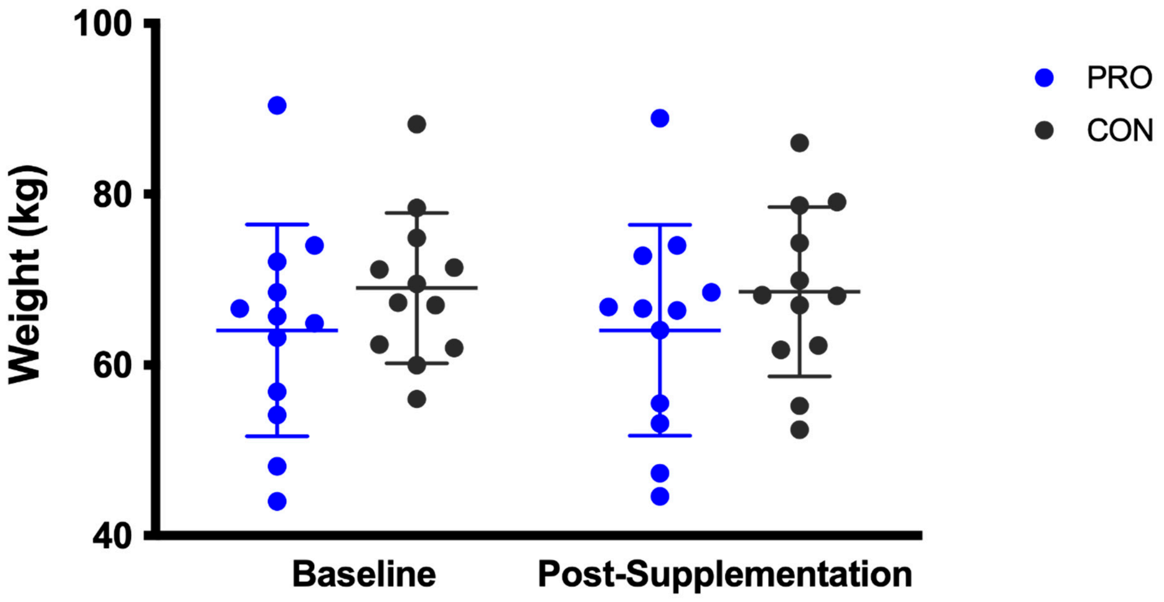

Figure S6. Participant Weight
Body weight was not different between groups and did not change over the course of the study period. Dot plot represents each data point with mean ± SD.
